# Transcriptome analysis of calcium hydroxide tolerance in *Enterococcus faecalis*

**DOI:** 10.3389/fmicb.2025.1551824

**Published:** 2025-04-30

**Authors:** Zhibo Xu, Haonan Ma, Xinmiao Jiang, Quzhen Baima, Yuqi Zhen, Shipeng Yang, Xiuping Meng

**Affiliations:** ^1^Department of Endodontics, Hospital of Stomatology, Jilin University, Changchun, China; ^2^School of Stomatology, Bengbu Medical University, Bengbu, China; ^3^Department of Periodontology, Hospital of Stomatology, Jilin University, Changchun, China

**Keywords:** *Enterococcus faecalis*, calcium hydroxide, tolerance, persistent apical periodontitis, transcriptomics

## Abstract

Calcium hydroxide (Ca(OH)_2_) is commonly used as a root canal disinfectant, but its effectiveness against *Enterococcus faecalis* is limited, likely due to the bacterium’s ability to tolerate it. This study aimed to investigate the underlying mechanism of *E. faecalis* tolerance to repeated exposure to Ca(OH)_2_. Initially, *E. faecalis* was exposed to Ca(OH)_2_ for 10 successive passages. The survival rate of the bacteria increased progressively, suggesting the development of tolerance to Ca(OH)_2_. Crystal violet staining revealed that calcium hydroxide-tolerant strains formed more robust biofilms compared to standard strains. To delve into the mechanisms of Ca(OH)_2_ tolerance in *E. faecalis*, RNA sequencing was employed for a preliminary investigation. Transcriptome sequencing identified 683 differentially expressed genes (DEGs) in the calcium hydroxide-tolerant strain, with 368 genes upregulated and 315 downregulated compared to the standard strain. Bioinformatics analysis revealed that the upregulated genes were associated with carbohydrate transport and metabolism, starch and sucrose metabolism, quorum sensing, aminoacyl-tRNA biosynthesis, and two-component systems signaling pathways. In contrast, the downregulated genes were primarily linked to lipoic acid metabolism, degradation of valine, leucine, and isoleucine, and the citric acid cycle (tricarboxylic acid cycle) signaling pathways. The findings suggest that prolonged exposure to Ca(OH)_2_ induces tolerance in *E. faecalis*. RNA sequencing further revealed that this tolerance involves multiple interconnected signaling pathways, providing a critical foundation for future research into therapeutic strategies for *E. faecalis* infections.

## 1 Introduction

*Enterococcus faecalis* (*E. faecalis*) is a Gram-positive, facultative anaerobic bacterium commonly found in refractory apical periodontitis (RAP). It is also associated with systemic diseases such as bacterial endocarditis, urinary tract infections, wound infections, and bacteremia (Gaeta et al., 2023; de Castro Leitão Godoy et al., 2023). RAP is a chronic oral infectious disease characterized by persistent inflammation, progressive destruction of alveolar bone, and impaired bone healing. Research shows that *E. faecalis* is the primary pathogen responsible for RAP. It has developed various survival strategies, leading to persistent infections both inside and outside the root canal system (Dai et al., 2020). Currently, root canal treatment is recognized as an effective approach for eliminating microbial infections and preventing reinfection (Siqueira and Lopes, 1999). The procedure of root cannal treatment includes three main steps: root canal preparation, root canal disinfection, and root canal filling. This therapy works by removing infected tissues, microorganisms, and their metabolic byproducts from the root canal system, followed by careful filling to prevent reinfection and promote the healing of surrounding tissues (Cosan et al., 2022; Mahfouz Omer et al., 2022). Calcium hydroxide (Ca(OH)_2_) is widely used in root canal disinfection due to its antimicrobial properties, ability to dissolve necrotic tissue, and promotion of hard tissue formation (Uzunoglu-Özyürek et al., 2018; Abe and Honda, 2023). The antimicrobial action of Ca(OH)_2_ is mainly due to the highly alkaline environment created by its dissociated OH^−^ ions. These ions cause lipid peroxidation, leading to phospholipid degradation and bacterial cell membrane destruction. The alkaline conditions also disrupt protein tertiary structures, inactivating proteins and causing metabolic disorders. Additionally, OH^−^ ions react with bacterial DNA, fragmenting it and impairing replication, thereby disrupting cellular functions (Mohammadi and Dummer, 2011).

However, studies indicate that 1 week after sealing a root canal with Ca(OH)_2_, the pH inside the canal is about 10.3 to 10.6 (Pedrinha et al., 2022). Ca(OH)_2_ demonstrates significant challenges in achieving complete eradication of *E. faecalis* within root canal systems. The intricate anatomy of root canals often prevents Ca(OH)_2_ from adequately penetrating narrow or curved regions. Additionally, the natural buffering capacity of dentin reduces the antimicrobial activity of Ca(OH)₂ within dentinal tubules, resulting in insufficient local drug concentrations. These conditions may promote *E. faecalis* tolerance to Ca(OH)_2_ by triggering the upregulation of specific genetic and proteomic pathways and encouraging biofilm formation on Ca(OH)_2_ surfaces (Flahaut et al., 1997; Distel et al., 2002). Clinical studies show that *E. faecalis* often persists in failed endodontic cases, displaying significant resistance to standard retreatment procedures (Evans et al., 2002). Post-treatment microbial analyses reveal that 8–30% of root canals treated with Ca(OH)_2_ still harbor viable microorganisms. Retreatment failure rates are strongly associated with the preoperative presence of *E. faecalis*, especially when detected during the obturation phase. These resilient bacteria survive within dentinal tubules, significantly reducing the success of endodontic treatments (Kayaoglu et al., 2009). Additionally, *E. faecalis* is known to develop multi-drug resistance, potentially exacerbated by the use of common disinfectants like chlorhexidine (Kitagawa et al., 2016; Wei et al., 2021). Although *E. faecalis* has been reported to survive the high pH environment created by Ca(OH)_2_, the specific survival mechanisms that enable this bacterium to tolerate exposure to Ca(OH)_2_ remain poorly understood.

*E. faecalis*, a frequent cause of persistent infections after root canal treatment, has gained attention for its ability to tolerate Ca(OH)_2_. Research indicates that this tolerance may be linked to biofilm formation, which acts as a physical barrier, and the regulation of pH homeostasis through proton pumps (Anija et al., 2021; Yang et al., 2024). Therefore, this study aimed to investigate whether *E. faecalis* develops increased tolerance to Ca(OH)_2_ following repeated exposure and to explore the molecular mechanisms involved using RNA sequencing techniques.

## 2 Materials and methods

### 2.1 Bacterial strains and growth conditions

*E. faecalis* (ATCC 29212) was used in this study. It was stored in a 50% glycerol solution at −80°C and initially cultured on brain heart infusion (BHI) agar (Oxoid, Basingstoke, Hampshire, United Kingdom) to obtain a monoclonal colony. Subsequently, the monoclonal colony was inoculated and incubated in BHI medium for 24 h at 37°C under aerobic conditions.

### 2.2 Ca(OH)_2_ adaptation development

The adaptation development of *E. faecalis* was conducted according to the modified method described by Huang et al. (2022). Initially, Ca(OH)_2_ was added to the BHI medium to create a saturated solution. After that, the freshly prepared saturated Ca(OH)_2_ solution was allowed to stand for 24 h, followed by centrifugation at a speed of 4,000 rpm for 10 min. The supernatant was then sterilized using a 0.22-μm pore size filter unit. Finally, the supernatant was diluted with BHI medium to obtain a Ca(OH)_2_ BHI medium with a pH of 10.4.

After a 24-h culture, *E. faecalis* was collected by centrifugation and resuspended in 10 mL of Ca(OH)_2_ BHI, achieving a final concentration of 1 × 10^8^ CFU/mL. Following a 24-h incubation period at 37°C, the pH value and colony forming units (CFU) were recorded, and the latter were logarithmically transformed.

**Figure 1:**
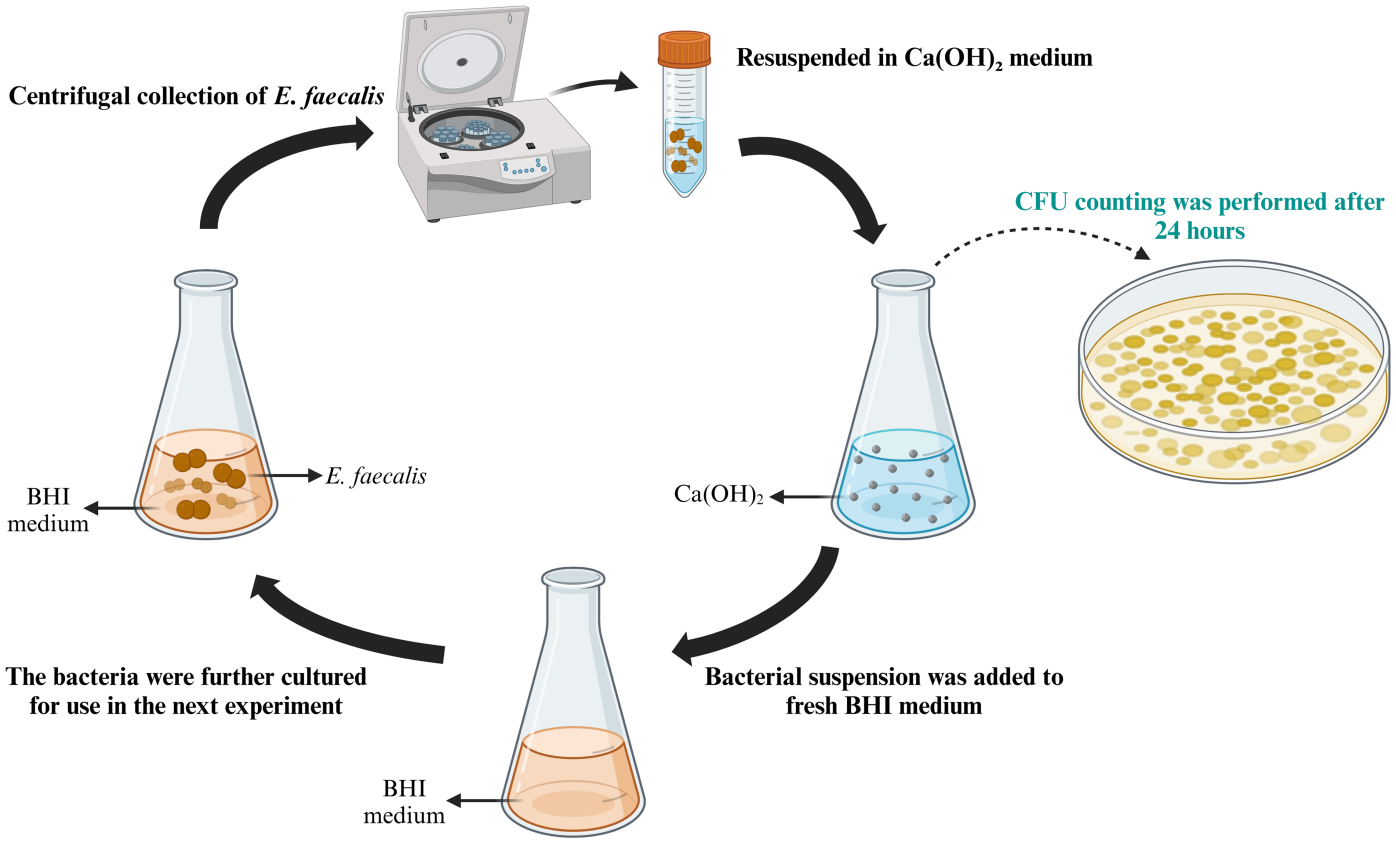
The process of tolerance development in *E. faecalis* exposed to Ca(OH)_2._

One milliliter of the residual bacterial suspension was combined with BHI medium and incubated for 24 h. The centrifuged bacteria were then resuspended in 10 mL of Ca(OH)_2_ BHI medium, adjusted to a concentration of 1 × 10^8^ CFU/mL for the next experiment. This procedure was repeated for 10 passages (Figure 1). Bacteria of the last passage with increased CFU was termed as the calcium hydroxide-tolerant strain, which was stored at −80°C for further experiments. The bacteria continuously cultured in BHI medium, as previously described, served as the control group.

### 2.3 Crystal violet assay

To facilitate biofilm formation under *in vitro* conditions, 1 mL of bacterial suspension in BHI medium with 1% sucrose was placed in a 24-well plate and incubated aerobically at 37°C for 48 h. The biofilm was rinsed twice with PBS to remove excess planktonic bacteria and fixed with 500 μL of 4% paraformaldehyde for 15 min. Following fixation, it was stained with 300 μL of 0.1% crystal violet (Aladdin, Shanghai, China) in the dark for 15 min. After washing twice with PBS to remove excess dye, 1 mL of 95% ethanol was added to each well to completely dissolve the crystal violet. The solution was then transferred to a 96-well plate to measure the optical density at 550 nm.

### 2.4 RNA extraction and sequencing

Bacteria were cultured in BHI medium for 24 h and collected by centrifugation at 5,000 rpm for 5 min at 4°C. Total bacterial RNA was extracted using the RNAiso Pure RNA Kit and digested with DNase I (Life Technologies Corporation, Gaithersburg, MD, United States). The NanoDrop spectrophotometer (Nano Drop 2000, Thermo Fisher Scientific, Waltham, MA, United States), Qubit 2.0 fluorometer (Q32866, Invitrogen, Carlsbad, CA, United States), and agarose gel electrophoresis were used to assess the degree of RNA degradation and to ascertain the absence of contamination. Only RNA that met the requisite quality standards could be utilized for subsequent library construction. The samples were quantified using TBS380 Picogreen (Invitrogen, Carlsbad, CA, United States), and the sequencing was performed on the Illumina HiSeq 2500 platform (Illumina, San Diego, CA, United States).

### 2.5 Quality control and filtering of raw reads

To ensure accurate analysis, it is essential to filter the raw reads. Initially, it is advisable to utilise FastQC (Version 0.11.2) to visually assess the quality of the sample’s sequencing data. Following this, Trimmomatic should be used to process the data, removing sequences with adapters and low quality in accordance with the following parameters: (1) removal of adapter sequences (adapters); (2) removal of sequences with fragment lengths <50 bp; (3) removal of sequences with N bases; (4) removal of bases with a quality score of less than 20 according to PHRED quality score.

### 2.6 Differentially expressed genes analysis

Differential expression analysis was performed using DESeq2 (McDermaid et al., 2019) (Version 1.12.4). Raw counts were normalized using the DESeq2 median of ratios method to correct for library size and compositional bias (Carbonetto et al., 2023). The normalized counts were then log2-transformed to stabilize variance prior to downstream analysis. RNA-seq count data were modeled using a negative binomial distribution to account for overdispersion, and differential expression was tested through generalized linear models (GLMs) implemented in DESeq2. Statistical significance was defined as an adjusted *p*-value (false discovery rate, FDR) <0.05 and |log2 fold change| ≥1 (Benjamini and Hochberg, 1995). All tests were two-tailed.

### 2.7 Functional and pathway enrichment analysis of DEGs

In order to gain a comprehensive understanding of the functions and metabolic pathways of differentially expressed genes (DEGs), a Gene Ontology (GO) annotation analysis was conducted on the DEGs. Additionally, the functional characteristics of the DEGs were analyzed through the Clusters of Orthologous Groups (COG) database, and an enrichment analysis of the DEGs in biological metabolic pathways was performed using the Kyoto Encyclopedia of Genes and Genomes (KEGG) database.

### 2.8 Statistical analysis

Data were processed and visualized using GraphPad Prism 9.0 (GraphPad Software, San Diego, CA, United States) and are presented as mean ± SD. A Student’s *t*-test was used to calculate *p*-values (^*^p < 0.05).

## 3 Results

### 3.1 Development of tolerance in *Enterococcus faecalis* to Ca(OH)_2_

**Figure 2:**
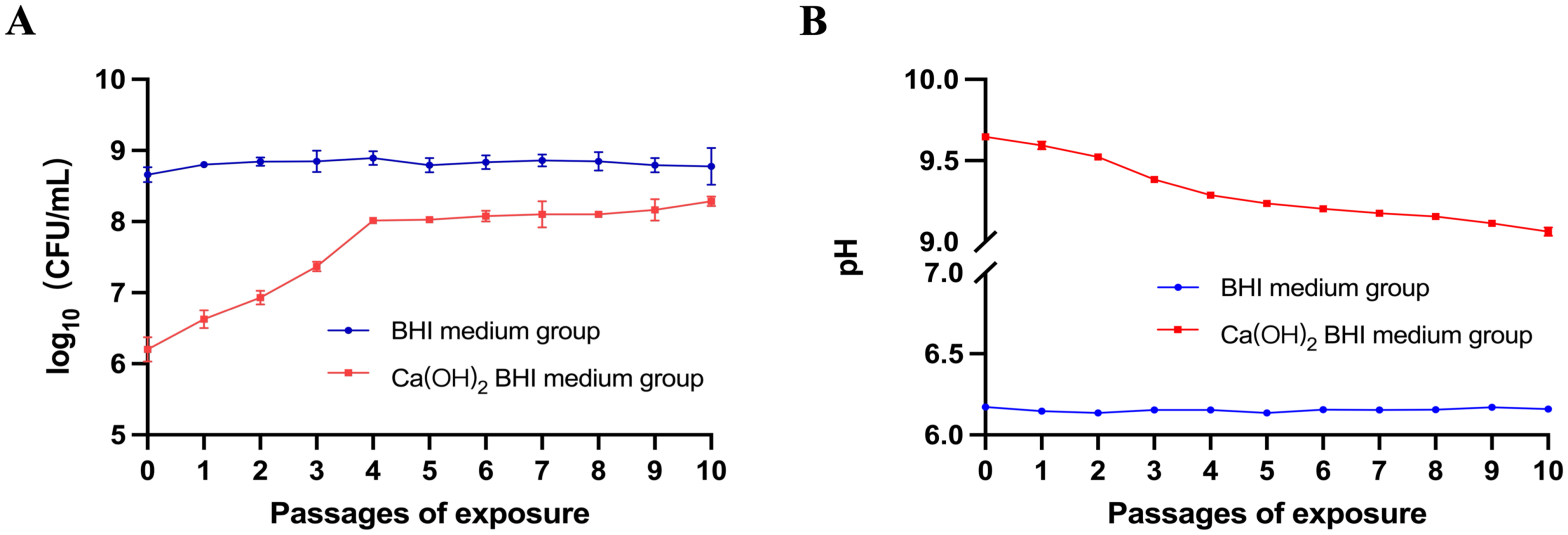
Study on *E. faecalis* tolerance to Ca(OH)_2_: **(A)** *E. faecalis* ATCC 29212 was exposed to Ca(OH)_2_ and regular BHI medium over 10 repeated passages, with survival rates recorded for each passage after 24 h. The red line represents survival in Ca(OH)_2_ BHI medium, and the blue line shows survival in regular BHI medium. **(B)** The experiment also measured the pH of the bacterial supernatant after each passage in both media. Data are presented as mean ± SD.

*E. faecalis* was exposed to Ca(OH)_2_ for 10 passages, and the bacterial survival rate gradually increased from passage 0 to passage 10. The viable bacterial numbers [mean CFU/mL (standard deviation)] increased from 1.67 × 10^6^ (5.77 × 10^5^) to 1.93 × 10^8^ (3.21 × 10^7^) and the pH decreased from 9.65 (0.02) to 9.06 (0.03). The control group exhibited minimal changes in CFU and pH. The CFU changed from 4.67 × 10^8^ (1.16 × 10^8^) to 6.67 × 10^8^ (3.21 × 10^8^) and the pH changed from 6.17 (0.01) to 6.16 (0.01) (Figures 2A,B). These results indicate that repeated exposure to Ca(OH)_2_ can effectively select for strains tolerant to Ca(OH)_2_. The 10th passage of bacteria exposed to Ca(OH)_2_ was designated as the *E. faecalis* calcium hydroxide-tolerant strain (*EF*-CHs).

### 3.2 Comparison of biofilm formation between *Enterococcus faecalis* and *EF*-CHs

**Figure 3:**
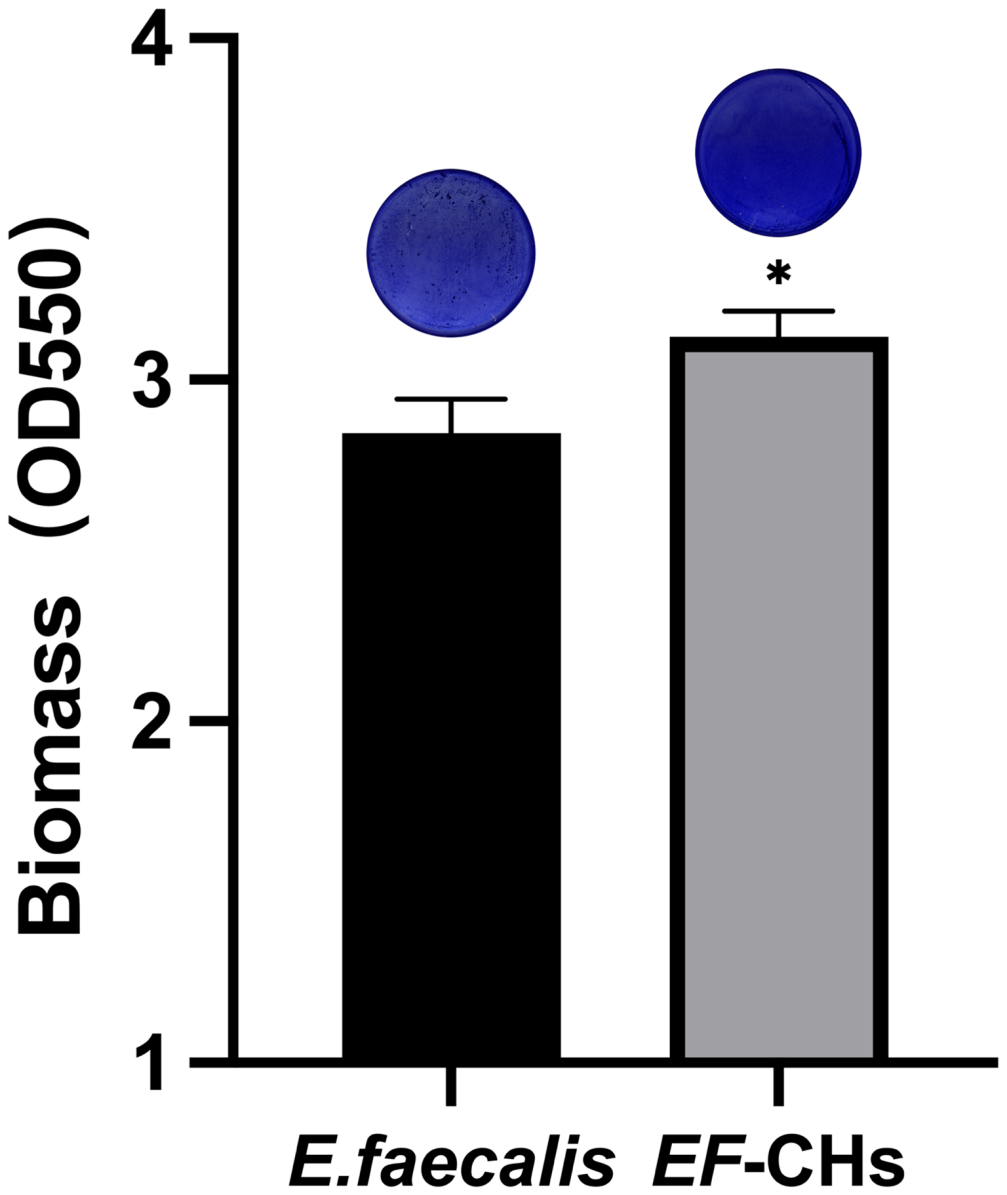
The crystal violet staining method was used to compare biofilm formation between *E. faecalis* and *EF*-CHs. A microplate reader measured the optical density of crystal violet dissolved in 95% ethanol at 550 nm. Higher values indicate greater biofilm formation. Results represent the average of three replicates and are shown as mean ± SD. ^*^p < 0.05.

The quantity of biofilm formation by the *E. faecalis* and *EF*-CHs was evaluated using a crystal violet staining assay. The results indicated that *EF*-CHs formed denser, more compact biofilms with a higher OD550 value than *E. faecalis* (Figure 3).

### 3.3 Sequencing data quality control and reference alignment analysis

To obtain high-quality sequencing data, it is essential to perform quality control and filter the original sequencing data. After removing adapter sequences and low-quality reads, these results indicate that the transcriptome dataset contains high-quality sequencing reads. The reference genome of *E. faecalis*, ATCC 29212 (NZ_CP008816.1), was selected from the NCBI database for analysis purposes. The read count, which indicates sequencing depth, exceeded 16 million for all samples in this study, ensuring adequate coverage of the transcriptome’s dynamic range (McDermaid et al., 2019). The Q20 ratio, which measures the proportion of bases with a quality score ≥20, exceeded 99.3% for all samples. This indicates exceptionally high raw data accuracy, reducing the risk of error in downstream analyses. The mapping rate, which measures how effectively sequencing data aligns to the reference genome, was ≥98.4% for standard strains and ≥93.1% for resistant strains. These values indicate strong compatibility between the sequencing data and the reference genome, providing a robust basis for differential gene expression analysis. The results demonstrate both the high quality of the sequencing data and the accuracy of genome measurements obtained from the strains under investigation. Table 1 provides detailed information on clean reads, PHRED quality scores, and successful mapping to the reference genome for both control and experimental groups.

**Table 1:** Quality of sequencing data and alignment to reference sequences.

Sample	*EF*-1	*EF*-2	*EF*-3	*EF*-CHs-1	*EF*-CHs-2	*EF*-CHs-3
Read count	17,804,890	18,713,394	17,882,364	18,904,432	16,266,162	21,026,742
Q20 ratio	99.42%	99.39%	99.37%	99.42%	99.31%	99.46%
Mapped	98.92%	98.75%	98.47%	96.56%	93.12%	97.06%

### 3.4 Transcriptome sequencing and clustering analysis

**Figure 4:**
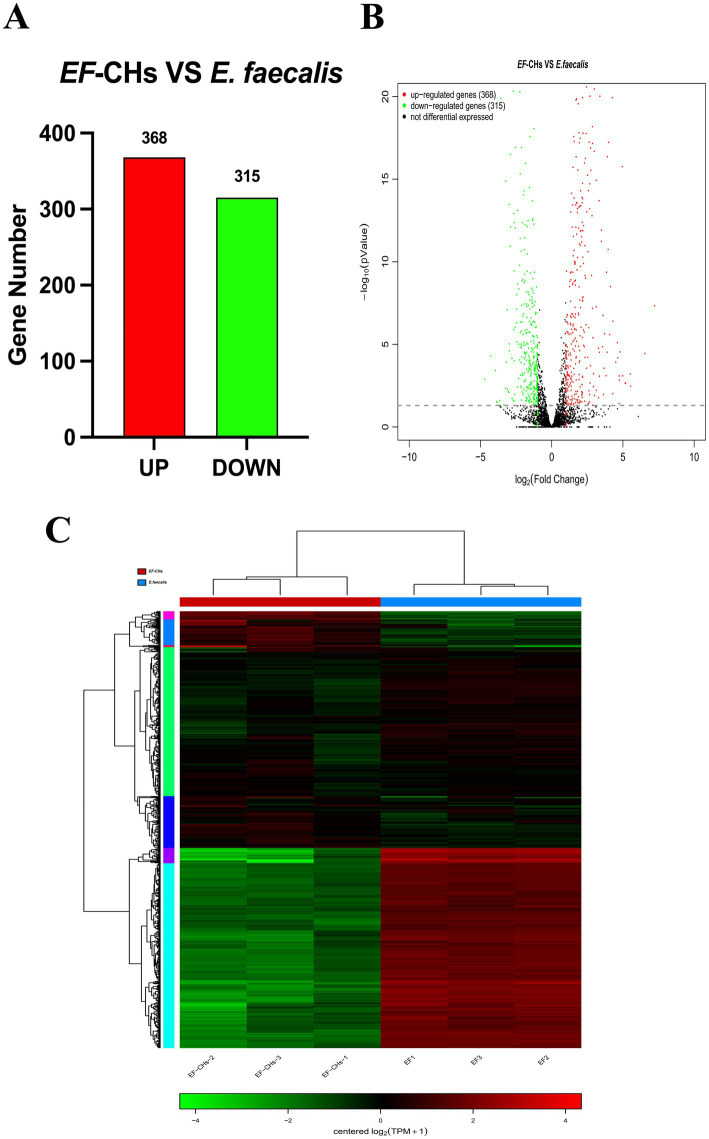
DEGs between the *EF*-CHs and *E. faecalis* groups were analyzed. **(A)** A volcano plot shows the distribution of DEGs, with red indicating up-regulated and green indicating down-regulated genes. **(B)** A histogram displays the number of up- and down-regulated genes. **(C)** A heat map illustrates DEG clustering, where each row is a gene and each column is a sample. Colors indicate gene expression levels, with red showing higher expression and green showing lower. Darker shades represent higher expression levels.

Significant transcriptional differences in 683 genes were observed between the *EF*-CHs and *E. faecalis* groups, with 368 genes upregulated and 315 downregulated in the *EF*-CHs group (Figures 4A,B). A pattern clustering analysis was then performed on the DEGs, resulting in a heat map of the clustered genes (Figure 4C). The heat map illustrates the expression patterns of both DEGs and samples. The results show that gene expression patterns are highly similar within each group but show significant variation between different groups. Additionally, the samples within each group are well-replicated. The data obtained is both credible and logical. Each square represents a DEG, where red indicates upregulated genes and green indicates downregulated genes. The color intensity corresponds to gene expression levels, with darker shades representing higher expression. The horizontal axis represents experimental samples, while the vertical axis shows clustering of the same gene across different samples. The two groups exhibit distinct gene expression patterns, characterized by consistent expression levels within each group and significant differences between the groups. The high reproducibility of samples within each group confirms the reliability, credibility, and consistency of the data.

### 3.5 GO functional annotation of DEGs

**Figure 5:**
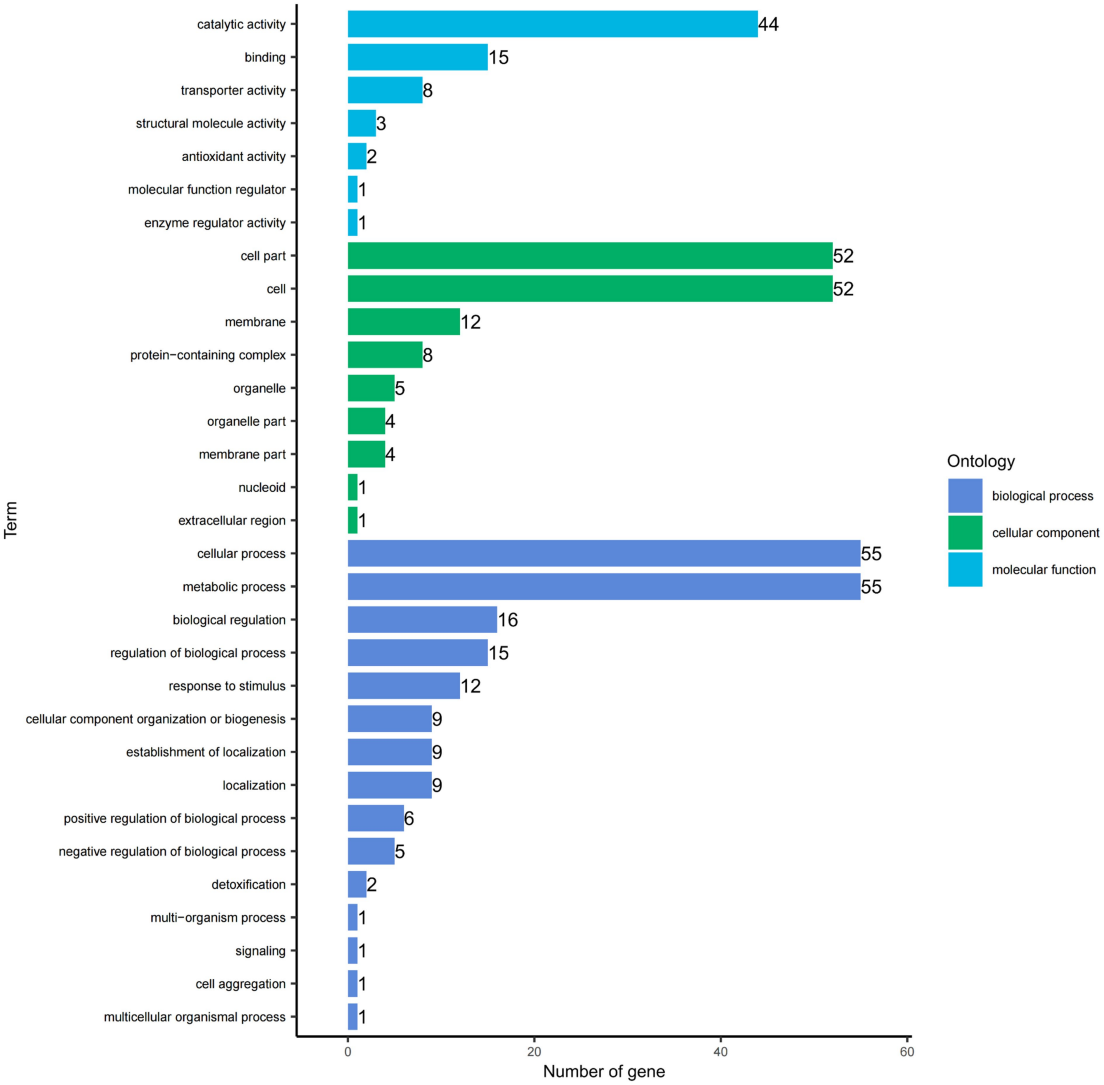
Gene Ontology (GO) functional categories of DEGs. The y-axis lists the functional categories, while the x-axis indicates the number of genes from the differential gene set in each category.

The GO functional annotation results indicate that the DEGs can be classified into three main categories: biological process (BP), cellular component (CC), and molecular function (MF). Within the biological process category, the highest number of DEGs are involved in metabolic processes (55 genes) and cellular processes (55). In the cellular component category, the most DEGs are associated with the cell (52) and cell part (52). For molecular function, the highest number of DEGs are involved in catalytic activity (44). Each DEG can be assigned to multiple functional categories (Figure 5).

### 3.6 Enrichment analyses of COG

**Figure 6:**
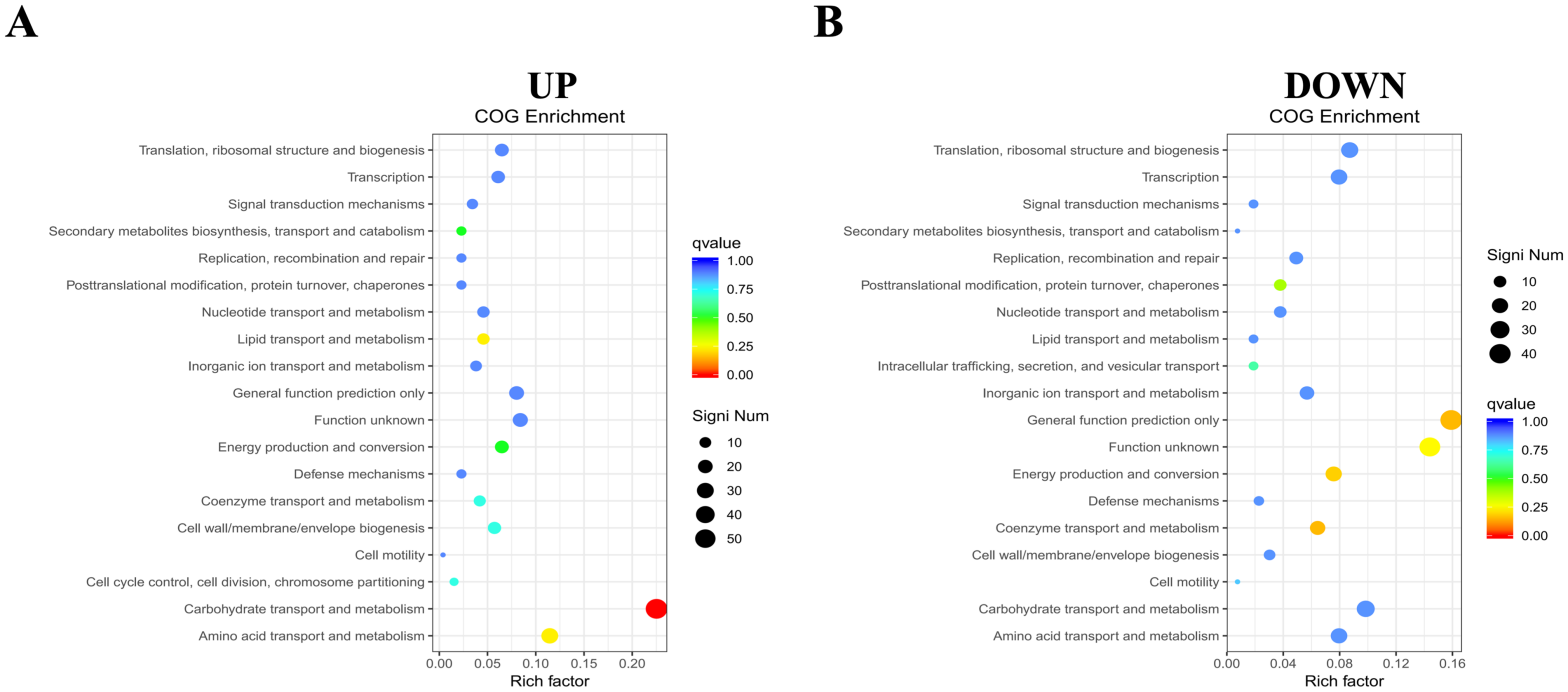
COG functional enrichment heatmap of DEGs. **(A)** Enrichment for up-regulated genes. **(B)** Enrichment for down-regulated genes. The y-axis shows functional annotations, and the x-axis shows the ratio of differentially expressed genes in each functional category to the total annotated genes in that category. The size of each point indicates the number of differentially expressed genes in a functional entry, while color intensity reflects the significance of enrichment.

To further analyze the biological functions associated with upregulated and downregulated differential genes, we performed COG enrichment analysis of the DEGs. A total of 368 upregulated DEGs were annotated into 19 COG functional categories. Only one functional category, carbohydrate transport and metabolism, was significantly enriched. The gene with the largest difference in this category was DR75_RS05265, predicted to encode the phosphotransporter system fructose transporter protein subunit IIA. Meanwhile, the 315 downregulated DEGs were also annotated into 19 functional categories, but none were significantly enriched (Figure 6).

### 3.7 KEGG pathway analyses of DEGs

**Figure 7:**
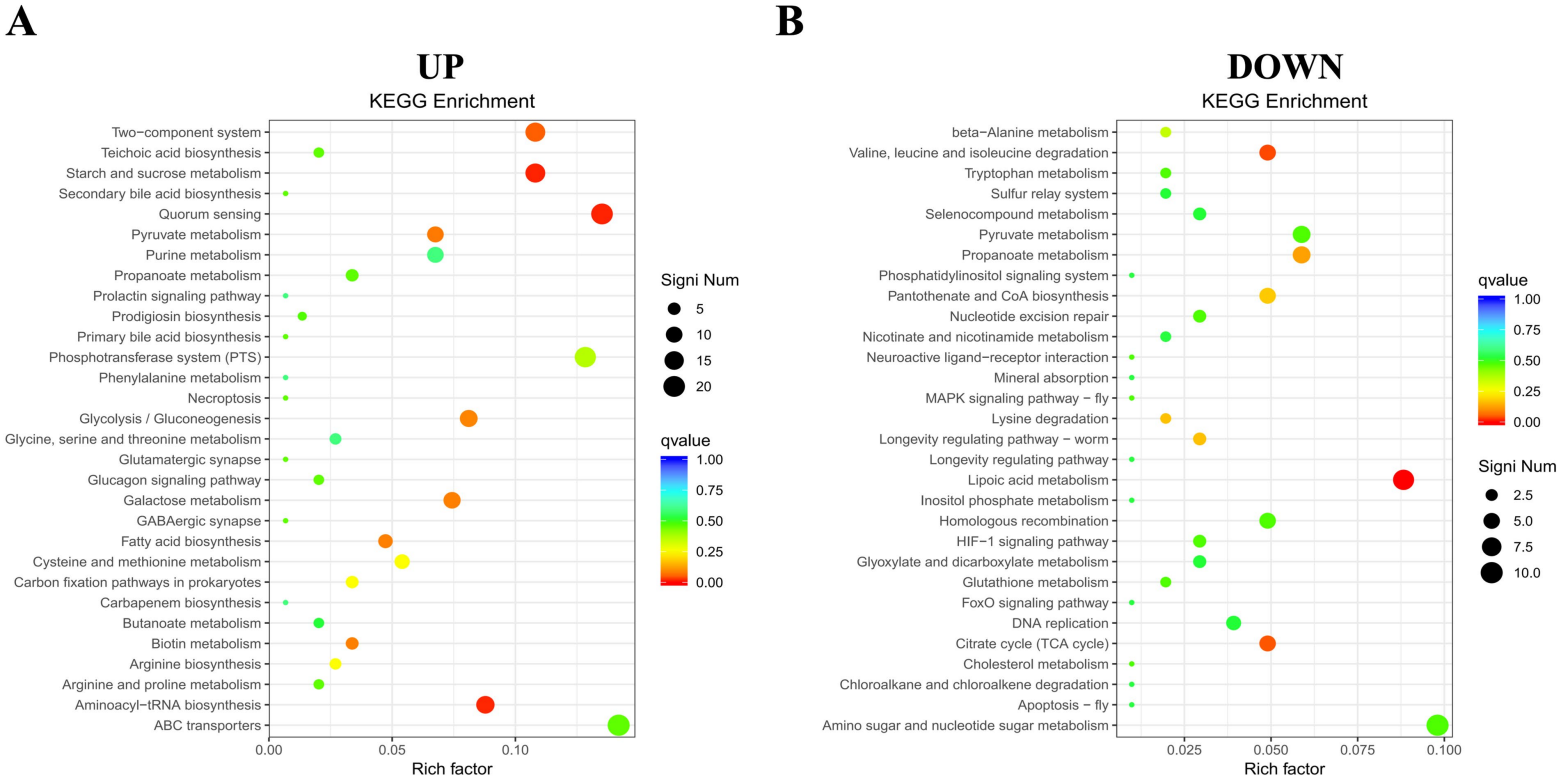
KEGG pathway enrichment map of DEGs. **(A)** Enrichment for up-regulated genes. **(B)** Enrichment for down-regulated genes. The y-axis displays functional annotations, while the x-axis indicates the ratio of DEGs in each pathway entry to the total annotated genes in that entry. Dot size reflects the number of DEGs in each pathway, and color intensity indicates the significance of the enrichment.

To gain a comprehensive understanding of the metabolic and signal transduction pathways involved in Ca(OH)_2_ tolerance in *E. faecalis*, KEGG pathway enrichment analyses were performed on both upregulated and downregulated DEGs. A total of 368 upregulated DEGs were assigned to 63 KEGG metabolic pathways, with four pathways showing significant enrichment. Only the top 30 enriched pathways are presented in the figure. The most significantly enriched pathways were starch and sucrose metabolism and quorum sensing, followed by aminoacyl-tRNA biosynthesis and the two-component system. Among the four significantly enriched pathways, the genes with the largest fold changes were DR75_RS03680, DR75_RS02045, *glyQ*, and DR75_RS00615. In contrast, among the downregulated DEGs, 315 were annotated to 77 KEGG metabolic pathways, with three pathways showing significant enrichment. The top 30 enriched pathways are listed below. These include lipoic acid metabolism, valine, leucine and isoleucine degradation, and the citric acid cycle (tricarboxylic acid cycle). Interestingly, the gene with the greatest fold change in all three pathways is *lpdA* (Figure 7 and Table 2).

**Table 2:** KEGG list of differentially expressed genes and the most significant differentially expressed gene in each pathway.

Up*/*Down	KEGG ID	Description	Most significant DEG	Potential roles in Ca(OH)_2_ tolerance
Up	ko00500	Starch and sucrose metabolism	DR75_RS03680	Biofilm formation
Up	ko02024	Quorum sensing	DR75_RS02045	Biofilm formation and drug resistance
Up	ko00970	Aminoacyl-tRNA biosynthesis	*glyQ*	Drug resistance
Up	ko02020	Two-component system	DR75_RS00615	Biofilm formation and Ca(OH)_2_ tolerance
Down	ko00785	Lipoic acid metabolism	*lpdA*	Reduced oxygen consumption and enhanced stress resistance
Down	ko00280	Valine, leucine and isoleucine degradation	*lpdA*	Reduced oxygen consumption and enhanced stress resistance
Down	ko00020	Citrate cycle (TCA cycle)	*lpdA*	Reduced oxygen consumption and enhanced stress resistance

## 4 Discussion

In the present study, following 24 h of culturing *E. faecalis* in a BHI medium supplemented with Ca(OH)_2_, we observed an initial decrease in the log CFU of *E. faecalis* from 8 to 6.2, which was subsequently followed by a gradual increase over four subsequent passages of *E. faecalis* culture in the same pH Ca(OH)_2_ medium. The log CFU of *E. faecalis* remained stable at approximately 8 after the fifth passage of culture and continued to remain stable until the tenth passage of culture. The findings suggest that *E. faecalis* maintains a stable and slowly proliferating state in the presence of Ca(OH)_2_, which is consistent with existing literature linking slow growth to tolerance (Zhang et al., 2022; Ordinola-Zapata et al., 2023). Coincidentally, some studies have demonstrated that the repeated application of Ca(OH)_2_ medication in root canal treatment as the potential to enhance the tolerance of *E. faecalis* towards it. In the study, the 10th passage of bacteria cultured under a BHI medium supplemented with Ca(OH)_2_ was designated as *EF*-CHs. Additionally, we observed a progressive decrease in the pH of the *E. faecalis* culture medium during successive passages in Ca(OH)_2_, indicating an enhanced capacity of *EF*-CHs to regulate the alkaline environment. This suggests that the tolerance of *E. faecalis* to Ca(OH)_2_ may be attributed to its evolutionary adaptation in acid production or resistance against alkali under alkaline conditions.

Biofilm density reflects the bacteria’s ability to secrete extracellular matrix components, such as polysaccharides and proteins, and indicates the complexity of their three-dimensional structure (Flemming, 2016). In this study, *EF*-CHs were observed to produce thicker and denser biofilms. Biofilm density is not only a quantitative morphological indicator but also a critical parameter for evaluating bacterial environmental adaptability and pathogenic potential. The dense biofilms formed by *EF*-CHs likely increase their tolerance to Ca(OH)_2_ by acting as a physical barrier. This structure restricts the penetration of OH^−^ ions, thereby minimizing direct bacterial damage. Furthermore, previous studies (Wang et al., 2019) have shown that high-density biofilms are positively associated with resistance to disinfectants. These biofilms hinder drug penetration and can activate bacterial resistance genes. Under alkaline stress, biofilms aid bacterial survival by regulating pH, often through the secretion of acidic metabolic byproducts (Zhang et al., 2022). Notably, biofilm persistence is closely linked to refractory apical periodontitis. Their high-density structure can prolong infection cycles and significantly complicate treatment (Dede et al., 2023).

To understand the mechanism of Ca(OH)_2_ tolerance in *E. faecalis*, we performed transcriptome sequencing on *E. faecalis* ATCC 29212 and *EF*-CHs. Sequencing quality is fundamental to transcriptome studies, as it directly determines the accuracy of subsequent analyses. A low Q20 ratio increases the base-calling error rate, which may lead to misestimation of gene expression levels or the identification of false-positive DEGs (Carbonetto et al., 2023). Similarly, low mapping rates, often caused by contamination or mismatches with the reference genome, can render large portions of data unusable and reduce statistical power (McDermaid et al., 2019). This study implemented a rigorous quality control process, including adapter trimming, removal of low-quality reads, and exclusion of short fragments, to minimize technical noise and ensure the data captures true biological signals. High-quality data serve as the foundation for advanced analyses such as differential gene expression and functional enrichment, directly influencing the reliability of mechanistic interpretations.

*E. faecalis* carries a range of intrinsic resistance genes and is capable of acquiring resistance genes due to its efficiency in exchanging mobile genetic elements encoding antibiotic resistance genes under stress conditions (van Harten et al., 2017). Comparative analysis revealed 683 significantly DEGs in *EF*-CHs, with 368 up-regulated and 315 down-regulated. GO functional annotation indicated that the most common categories were metabolic processes, cellular processes, and catalytic activities, consistent with previous findings on *E. faecalis* under alkaline stress (Ran et al., 2015).

A COG enrichment analysis was conducted to further explore the biological functions of DEGs. This analysis revealed significant enrichment in carbohydrate transport and metabolism among up-regulated genes, with no significant enrichment among down-regulated genes. Research indicates that carbohydrate transport and metabolism enhance bacterial ability to obtain carbohydrates from the environment, aiding colonization and biofilm formation (Vebø et al., 2009; Koropatkin et al., 2012; Grand et al., 2020). The gene DR75_RS05265, which showed the highest differential expression, encodes a fructose transport protein subunit IIA of the phosphate transport system, essential for sugar uptake and phosphorylation in bacteria (Keffeler et al., 2021). This system helps bacteria endure harsh conditions and improve survival (Peng et al., 2017).

To deepen our understanding of metabolic and transduction pathways involved in Ca(OH)_2_ tolerance in *E. faecalis*, we conducted a KEGG enrichment analysis. This revealed significant enrichment in four metabolic pathways among up-regulated genes: starch and sucrose metabolism, quorum sensing, aminoacyl-tRNA synthesis, and the two-component system. Among down-regulated genes, significant enrichment was found in three pathways: lipoic acid metabolism, valine, leucine, and isoleucine degradation, and the citric acid (TCA) cycle.

Up-regulated genes in the *EF*-CHs group showed enhanced starch and sucrose metabolism, aligning with previous COG enrichment results on carbohydrate transport and metabolism. The gene DR75_RS03680, which encodes *α*-glucosidase, showed the highest level of differential expression. This enzyme facilitates the breakdown of complex sugars into monosaccharides. During this process, the acidic metabolic byproducts generated can partially neutralize the extracellular alkaline environment, thereby mitigating the damage caused by OH^−^ to cells. Additionally, monosaccharides can serve as precursors for the synthesis of biofilm matrix components, strengthening the stability of the polysaccharide backbone in the biofilm. This reinforcement improves the physical barrier function of the biofilm, restricts Ca(OH)_2_ penetration, and ultimately promotes bacterial pathogenicity and biofilm formation (Zhang et al., 2011; Moye et al., 2014). Additionally, previous experiments indicated that the biofilm formation capacity of the *EF*-CHs group was augmented compared to the *E. faecalis* group, suggesting that *E. faecalis* may develop Ca(OH)_2_ tolerance through enhanced biofilm formation.

Quorum sensing (QS) is an essential bacterial communication system that enables the coordination of collective behaviors through the secretion and detection of signaling molecules, known as autoinducers. When bacterial population density increases and the concentration of signaling molecules reaches a threshold, specific genes are activated, regulating processes such as biofilm formation, virulence factor release, and antibiotic resistance (Bassler and Losick, 2006; Wang et al., 2019). The ABC transporter permease encoded by the highly expressed gene DR75_RS02045, as part of the ABC transport system, is involved in transmembrane transport. This transporter regulates the expression of multidrug efflux pumps, accelerates OH^−^ efflux, and alleviates intracellular alkaline stress. Additionally, it has been demonstrated that this transporter plays an active efflux role in *E. faecalis* and is associated with bacterial antibiotic resistance (Hürlimann et al., 2016; Zhu et al., 2022). This suggests that Ca(OH)_2_ tolerance in *E. faecalis* may be connected to the quorum sensing system and ABC transporter proteins.

The gene *glyQ*, encoding the glycyl-tRNA ligase subunit *α*, showed the largest differential expression in the aminoacyl-tRNA anabolic pathway, significantly up-regulated in the *EF*-CHs group. This enzyme catalyzes the synthesis of glycyl-tRNA, essential for glycine insertion into proteins, and also synthesizes dinucleotide polyphosphates involved in cellular regulation (Yu et al., 2023). Evidence suggests *glyQ* as a potential drug target for drug-resistant bacteria, highlighting its link to bacterial drug resistance (Fereshteh et al., 2023; Noori Goodarzi et al., 2023).

The two-component system (TCS), the final significantly enriched pathway among up-regulated genes, plays a pivotal role in mediating drug resistance and environmental adaptation in *E. faecalis*. As a conserved bacterial signaling mechanism, TCS enables rapid perception of extracellular stimuli and transcriptional regulation of stress-responsive genes, facilitating survival under dynamic host or environmental conditions (Todd Rose et al., 2023; Zhang et al., 2023). This system is essential for preserving cell envelope integrity by coordinating membrane protein synthesis and stress response pathways, which are critical for bacterial persistence and virulence (Kellogg et al., 2017). Recent studies show two-component system associated with glycosyltransferases activity, EPS synthesis, and biofilm formation in *E. faecalis* (Wu et al., 2019). Inhibition of this system reduces polysaccharide production, impairs biofilm formation, and increases sensitivity to Ca(OH)_2_, aligning with our findings.

Among down-regulated genes, significant enrichment was found in three metabolic pathways, with *lpdA* showing the largest differential expression. The gene *lpdA* encodes thiooctylamide dehydrogenase, a component of the 2-ketoglutarate dehydrogenase (2-KGDH) and pyruvate dehydrogenase (PDH) complexes in the TCA cycle. It oxidizes protein-bound lipoic acid during decarboxylation of 2-ketoglutarate and pyruvate (Hassan and Cronan, 2011). Studies on lpdA-deficient *E. coli* show reduced oxygen consumption and enhanced survival and stress resistance (Gonidakis et al., 2010). It is postulated that *lpdA* downregulation in *E. faecalis* may similarly enhance Ca(OH)_2_ tolerance.

This study demonstrates that the tolerance of *E. faecalis* to Ca(OH)_2_ is closely related to its biofilm formation capacity. The biofilm formation ability of *EF*-CHs was significantly enhanced, with upregulated genes primarily enriched in starch and sucrose metabolism, quorum sensing, and two-component systems. While previous research has shown that *E. faecalis* tolerates alkaline environments through biofilm formation (Kayaoglu et al., 2009), the mechanisms by which prolonged Ca(OH)_2_ exposure modulates metabolic pathways to enhance biofilm formation remain unclear. This study is the first to reveal that the sustained upregulation of starch and sucrose metabolism genes may facilitate biofilm synthesis by supplying monosaccharide precursors (Zhang et al., 2011). Additionally, the activation of two-component systems, in coordination with quorum sensing, regulates extracellular polysaccharide secretion to construct a denser biofilm barrier (Wu et al., 2019). By integrating starch and sucrose metabolism, quorum sensing, and two-component systems, this study suggests that Ca(OH)_2_ tolerance results from the synergistic action of multiple pathways rather than the regulation of a single gene. These findings provide novel insights into resistance mechanisms against non-antibiotic disinfectants, filling a critical gap in understanding metabolic reprogramming and biofilm formation under alkaline stress.

**Figure 8:**
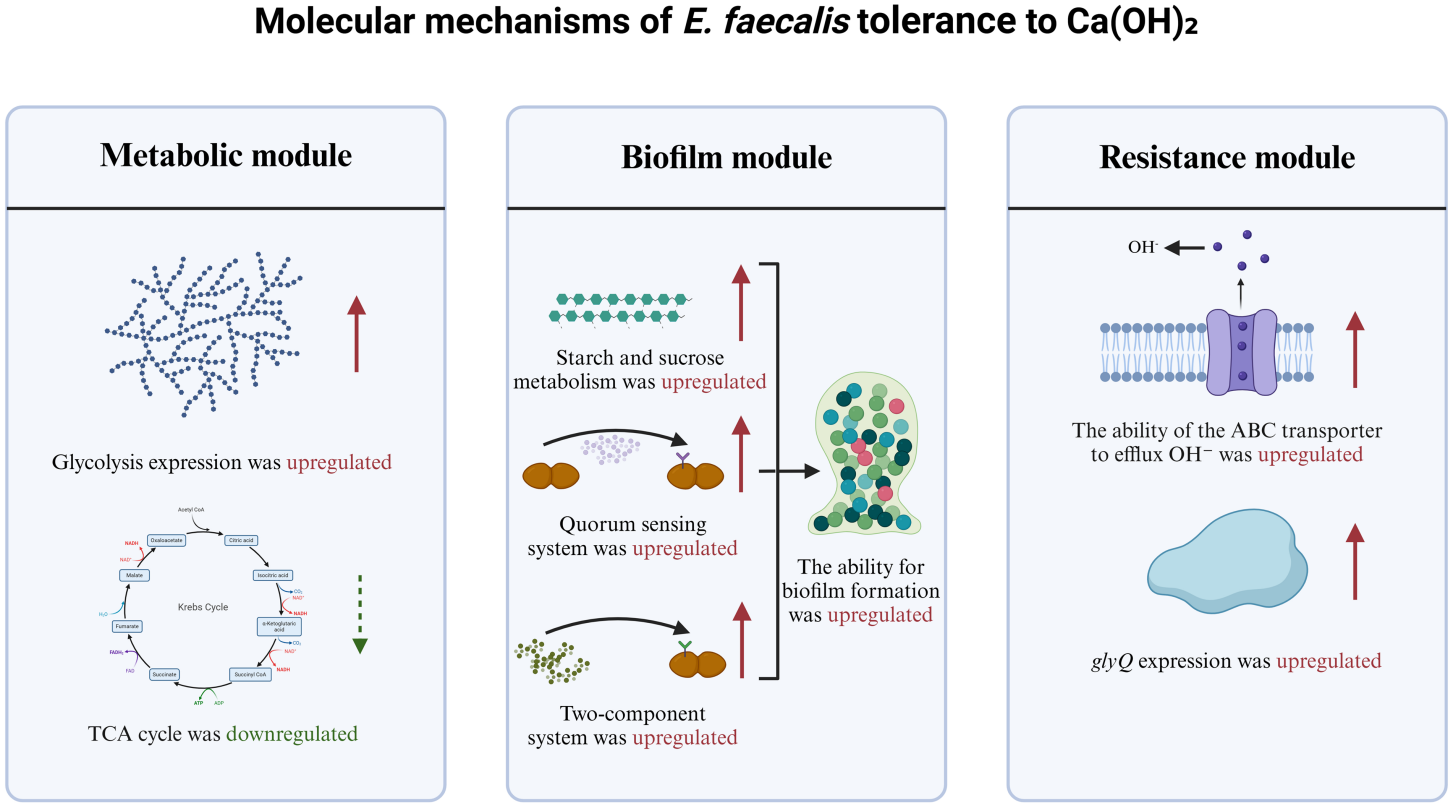
Molecular mechanisms of *E. faecalis* tolerance to Ca(OH)_2_.

Previous research has shown that bacteria suppress aerobic metabolism under nutrient-limited or stress conditions to conserve energy (Gonidakis et al., 2010). However, it remains unclear whether Ca(OH)_2_ tolerance relies on a similar strategy. This study is the first to reveal that *E. faecalis*, under prolonged Ca(OH)_2_ exposure, suppresses the TCA cycle while enhancing glycolysis to rapidly produce energy. This metabolic shift may represent a core strategy for tolerating high pH environments, complementing recent findings on alkaline adaptation in *E. coli* (Zhu et al., 2022). While the multidrug resistance of *E. faecalis* has been widely studied (van Harten et al., 2017), the mechanisms coordinating gene expression for Ca(OH)_2_ tolerance remain unexplored. Moreover, the upregulation of *glyQ* may support cellular homeostasis by facilitating protein synthesis (Yu et al., 2023), while ABC transporters likely mediate OH^−^ ions efflux or signaling molecule transmission (Hürlimann et al., 2016). This multi-pathway synergy provides a novel perspective on resistance mechanisms against non-antibiotic disinfectants, expanding our understanding of bacterial stress adaptation mechanisms (Figure 8).

This study has several limitations that should be acknowledged. First, the *in vitro* model used in this research simplifies the clinical complexity of root canal environments. For example, the absence of host immune factors may overestimate bacterial survival rates (de Castro Leitão Godoy et al., 2023). Second, the study focused exclusively on a single strain, ATCC 29212. While its complete genome data facilitates comparisons with sequencing results, the exclusion of other clinical isolates limits the broader applicability of the findings. Clinical isolates often exhibit genetic variability, including the presence of virulence genes that enhance biofilm compactness (Dede et al., 2023). Future research should validate these mechanisms using diverse clinical strains and incorporate *in vivo* models to better simulate clinical conditions.

In summary, this study provides the first comprehensive analysis of the global transcriptional responses of *E. faecalis* to prolonged exposure to Ca(OH)_2_. By conducting 10 passages of continuous exposure experiments, we uncovered the bacterium’s dynamic adaptation process, transitioning from initial stress to stable tolerance. Additionally, we identified 683 key DEGs, forming a robust foundation for future research. The identified key genes and pathways present promising targets for developing innovative anti-biofilm strategies. For instance, inhibitors designed to target critical enzymes involved in starch and sucrose metabolism, quorum sensing mechanisms, and two-component regulatory systems could be explored. These inhibitors may enhance the antibacterial efficacy of Ca(OH)_2_ by disrupting biofilm matrix synthesis. Furthermore, they may work synergistically with established root canal disinfection methods, including combination therapies with chlorhexidine (Mahfouz Omer et al., 2022). In addition, in the context of resistance monitoring, the significantly upregulated genes in *EF*-CHs could serve as biomarkers for detection. These DEGs may act as molecular indicators for assessing the resistance of clinical isolates, offering a scientific basis for designing personalized antibacterial therapies.

## 5 Conclusion

This study confirmed that *E. faecalis* develops tolerance after repeated exposure to Ca(OH)_2_ and successfully induced an *E. faecalis* calcium hydroxide-tolerant strain. The acid-producing and biofilm-forming abilities of the *E. faecalis* and the *EF*-CHs were compared by pH assay and crystal violet staining. Further transcriptome sequencing revealed that the upregulation of starch and sucrose metabolism, group sensing, aminoacyl-tRNA biosynthesis, and two-component system pathways, as well as the downregulation of lipoic acid metabolism, degradation of valine, leucine, and isoleucine, and the citric acid cycle (tricarboxylic acid cycle) pathways, may mediate the development of Ca(OH)_2_ tolerance in *E. faecalis* compared to the control group. These findings enhance our understanding of the mechanisms behind Ca(OH)_2_ tolerance in *E. faecalis*.

## Data Availability

The raw data supporting the conclusions of this article will be made available by the authors, without undue reservation.
